# Neumonía por COVID-19 en niños: De su etiología a su manejo

**DOI:** 10.1159/000516059

**Published:** 2021-04-12

**Authors:** Giuseppe Fabio Parisi, Cristiana Indolfi, Fabio Decimo, Salvatore Leonardi, Michele Miraglia del Giudice

**Affiliations:** 1 ^a^Departamento de Medicina Clínica y Experimental, Universidad de Catania, Catania, Italia; 2 ^b^Departamento de Mujeres, Niños y Cirugía Especializada, Universidad de Campania «Luigi Vanvitelli», Nápoles, Italia

**Keywords:** COVID-19, Niños, Neumonía, SARS-CoV2, Tratamiento

## Abstract

El COVID-19 es menos serio en niños que en adultos. Sin embargo, las afecciones respiratorias dominan el cuadro clínico de pacientes hospitalizados por COVID-19, aun en niños. En algunas series de casos, el deterioro del estado clínico, donde la disnea, la cianosis y el inicio del síndrome de dificultad respiratoria aguda (SDRA) emergieron ∼8–10 días después del inicio de la infección por SARS-CoV-2, pudo progresar rápidamente hasta la falla multiorgánica y la muerte. Esta revisión tiene como objetivo evaluar las características de la neumonía por COVID-19 en poblaciones pediátricas, comenzando con su etiología y sus mecanismos patológicos, para cerrar con su manejo clínico.

## Introducción

A finales de diciembre de 2019, un nuevo coronavirus que se originó en la ciudad china de Wuhan comenzó a extenderse rápidamente por todo el mundo [1]. A inicios de 2020, el Comité Internacional de Taxonomía de Virus lo denominó Coronavirus 2 del Síndrome Respiratorio Agudo Grave (SARS-CoV-2) [2]. El SARS-CoV-2 es el agente causal de la enfermedad COVID-19, abreviatura elegida por la Organización Mundial de la Salud (OMS). En otras palabras, SARS-CoV-2 es el agente etiológico, mientras que COVID-19 es la enfermedad [3].

El espectro clínico del COVID-19 es amplio, y varía desde formas completamente asintomáticas hasta aquellas caracterizadas por dificultad respiratoria grave que requiere cuidados intensivos. El SARS-CoV-2 causa una infección viral aguda tanto en el tracto respiratorio superior como en el inferior, con un periodo de incubación de 1 a 15 días (3–7 días en promedio). Los síntomas más comunes de COVID-19 incluyen fiebre, tos, dolor de garganta, cefalea, astenia, diarrea y vómito (4).

Hay evidencia abundante en la literatura de que el COVID-19 es menos serio en niños que en adultos [5, 6, 7, 8, 9, 10]. Lu y cols. encontraron que los principales síntomas en 171 niños con COVID-19 fueron tos (48.5%), faringitis (46.2%), fiebre (41.5%), diarrea (8.8%) y vómito (6.4%); sólo 2.3% de los casos experimentaron desaturación tras el ingreso al hospital, mientras que 15.8% fueron asintomáticos [9]. Las anomalías olfativas y gustativas características de los casos adultos de COVID-19 son raras en la población pediátrica [11, 12].

Hasta el momento, no está clara aún la causa subyacente de la menor incidencia y patogenicidad de la infección por SARS-CoV-2 en niños. Aunque esta menor incidencia y morbilidad se atribuyeron a una exposición reducida y a la menor presencia de factores de riesgo durante la fase inicial de la pandemia, hoy está claro que los factores biológicos que intervienen en la patogénesis de la infección y en la respuesta inmune podrían tener un papel protector en niños contra las manifestaciones clínicas más agresivas que vemos en los adultos [13].

Las afecciones respiratorias dominan el cuadro clínico de pacientes hospitalizados por COVID-19. En algunas series de casos, el deterioro del estado clínico, donde la disnea, la cianosis y el inicio del síndrome de dificultad respiratoria aguda (SDRA) emergieron ∼8–10 días después del inicio de la infección por SARS-CoV-2, pudo progresar rápidamente hasta la falla multiorgánica y la muerte [14]. En una serie de casos pediátricos de COVID-19, 30.8% de los pacientes presentaron dificultad respiratoria que requirió suplementación de oxígeno, y 23.1% ingresaron en la unidad de cuidados intensivos (UCI) por disfunción orgánica [15]. En otra serie de casos que incluyó 41 niños hospitalizados por COVID-19, 11 de ellos presentaron lesiones pulmonares compatibles con un cuadro de neumonía intersticial [16]. Adicionalmente, en una de las series pediátricas más extensas publicadas hasta ahora, que estudió 585 niños con infección por SARS-CoV2, 8% requirieron ingresar a la UCI, y 4% necesitaron ventilación mecánica [17].

Aunque el cuadro clínico en poblaciones pediátricas es más complejo, la gravedad de la infección puede clasificarse clínicamente como asintomática, leve, moderada, grave o crítica [18, 19] (Tabla 1). Esta clasificación indica que incluso los pacientes pediátricos pueden experimentar manifestaciones graves de la patología, que deben atenderse tan pronto como sea posible, para limitar la progresión de la enfermedad.

Esta revisión tiene como objetivo evaluar las características de la neumonía por COVID-19 en poblaciones pediátricas, comenzando con su etiología y sus mecanismos patológicos, para cerrar con su manejo clínico.

## Métodos

En esta revisión se emplearon PubMed y Science Direct para localizar artículos con resumen disponible en inglés (por lo menos), utilizando las siguientes palabras clave: (1) COVID-19 en niños; (2) coronavirus en niños; (3) neumonía por COVID-19; (4) SARS-CoV-2 en niños; (5) neumonía por SARS-CoV-2, y (6) estudios de imagen en COVID-19. Se revisaron los resúmenes de los artículos para determinar si eran apropiados para el tema. También revisamos las referencias en los artículos seleccionados, y leímos por entero los artículos que se consideraron relevantes.

## Epidemiología

El virus SARS-CoV-2 se transmite mediante pequeñas gotas y a través de contacto directo o indirecto con objetos infectados[ 1]. Aún no está claro el tiempo durante el cual el virus permanece activo en las superficies, pero se han encontrado valores ∼48–72 h en plástico y acero, y ∼4–8 h en cobre y cartón [20]. La convivencia con pacientes sintomáticos o asintomáticos es la principal fuente de contagio para poblaciones pediátricas [21], pero dada la frecuencia de formas paucisintomáticas en poblaciones pediátricas, es muy probable que los niños sean vectores frecuentes de infección para adultos y ancianos. La positividad en la reacción en cadena de la polimerasa con transcripción inversa (RT-PCR) para SARS-CoV-2 en las heces de infantes y niños por varias semanas, incluso después de un hisopado nasofaríngeo negativo [22], podría indicar que las heces representan un medio adicional de transmisión del virus.

Sin embargo, puesto que no se ha demostrado el crecimiento del virus en cultivo fecal − y por tanto su viabilidad en las heces −, se requieren estudios adicionales para definir una posible ruta fecal-oral para la transmisión del virus. De manera similar, se ha explorado la transmisibilidad materno-fetal del virus desde el inicio de la epidemia. Un primer reporte de nueve mujeres con COVID-19 en el tercer trimestre de embarazo confirmó la ausencia de SARS-CoV-2 en el líquido amniótico, la sangre del cordón y la leche materna [23]. Más recientemente, la transmisión materno-fetal se confirmó en tres infantes (tasa de transmisión: 9%) nacidos de una madre positiva; uno de estos infantes inició con síntomas respiratorios en las primeras 48 h de vida [24]. Sin embargo, un análisis retrospectivo con una cohorte mayor, que involucró a 101 infantes nacidos de 100 madres positivas para SARS-CoV-2 no mostró transmisión vertical en ninguno de ellos [25].

## Patogénesis del daño pulmonar

**Fig. 1:**
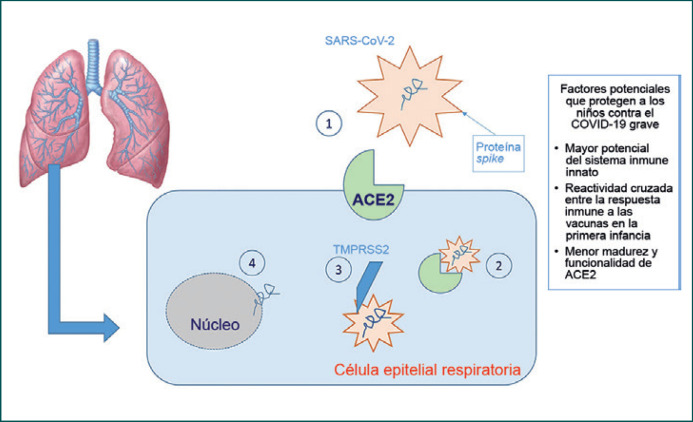
Representación gráfica de la interacción virus-huésped y las razones por las que los niños son menos afectados. (1) Unión entre la proteína *spike* del SARS-CoV2 y los receptores ACE2; (2) transporte del complejo receptor ACE2/SARS-CoV-2 al interior de la célula; (3) corte de la proteína *spike* por la proteasa TMPRSS2; (4) liberación del RNA viral dentro de la célula.

Cuando el SARS-CoV-2 entra en las vías respiratorias de una persona recientemente infectada, la proteína viral S (proteína *spike*) se une con gran afinidad al receptor transmembranal de la enzima convertidora de angiotensina 2 (ACE2) que se encuentra en las membranas apicales de las células epiteliales respiratorias, principalmente en los neumocitos de tipo II. Posteriormente, el receptor ACE2 y el SARS-CoV-2 son transportados al interior de la célula; la proteasa TMPRSS2 corta a la proteína S, induciendo la liberación del RNA viral dentro de la célula y, de ese modo, permite su replicación (Figura 1). Luego, el receptor ACE2 se separa por la acción de la enzima convertidora del factor de necrosis tumoral alfa (TACE o ADAM17), una metaloproteasa que permite la liberación del ectodominio de ACE2 (definido como ACE2 soluble) hacia el espacio extracelular. El ACE2 soluble es enzimáticamente activo, y parece ser capaz de unirse al SARS-CoV-2. Esto llevó a la especulación de que la administración de ACE2 humano recombinante podría reducir la inflamación secundaria a la acción del SARS-CoV-2 [26].

La respuesta inmune inducida por la infección por SARS-CoV-2 se caracteriza por la existencia de dos fases: una fase inicial inmunoprotectora y una fase de activación de la tormenta de citocinas, que conduce a una manifestación clínica más grave. En la primera fase, una robusta respuesta adaptativa puede controlar al virus y bloquear el progreso de la inflamación. Si el sistema inmune fracasa al controlar esta fase, el daño celular en órganos con altas concentraciones de ACE2, especialmente en los neumocitos, avanza con la liberación de citocinas y quimiocinas (IL-6, IL-10 e interferón), y el reclutamiento de células inflamatorias, que median el daño pulmonar y la progresión hacia la SDRA [27]. Xu y cols. encontraron evidencia de daño alveolar difuso con descamación de neumocitos, formación de membrana hialina y la presencia de células fibromixoides con infiltración linfocitaria intersticial, durante el examen histopatológico de un paciente que murió por COVID-19 [28]. De hecho, desde un punto de vista clínico, el SARS-CoV-2 causa neumonía intersticial.

Una de las posibles complicaciones de esta inflamación «exagerada» es el síndrome inflamatorio multisistémico pediátrico (PIMS) o síndrome inflamatorio multisistémico de la niñez (MIS-C), que ocurre cuando la inflamación se vuelve generalizada. Esto podría parecer una reacción posinmunológica causada por anticuerpos IgG no-neutralizantes, agravada por la tormenta de citocinas, que causa una inflamación generalizada similar a la enfermedad de Kawasaki atípica o al síndrome de choque tóxico [29].

### ¿Por qué los niños se ven menos afectados?

Se han propuesto muchas explicaciones para el hecho de que los niños parecen ser afectados con menor frecuencia y tener manifestaciones más leves de COVID-19; sin embargo, siguen siendo suposiciones dada la falta de evidencia científica al respecto.

La respuesta inmune de los niños difiere de la de los adultos, que se deteriora progresivamente con la edad, de manera que los niños en edad preescolar tienen un repertorio de células inmunes 5–10 veces mayor que el de un individuo de 50 años, y 20 veces mayor que el de un paciente de 80 años. Aún no se sabe hasta qué grado esto puede tener un papel en mitigar la propagación del virus y en la cascada de señalización de citocinas que desencadena el SARS-CoV-2, puesto que ambas se relacionan con efectos graves en la edad adulta [30].

También se ha propuesto una reactividad cruzada entre la respuesta inmune a las vacunas de la primera infancia − especialmente la vacuna triple viral − y la respuesta al SARS-CoV-2. Sin embargo, hasta la fecha no han surgido evidencias claras que apoyen esta propuesta, y se reportan casos paucisintomáticos aun en niños no-vacunados. En una extensa serie clínica pediátrica que incluyó 2143 niños se reportó una tasa de casos graves y críticos de 5.9%, y solamente una muerte [31]. Shekerdemian y cols. reportaron una tasa de mortalidad de 4.2% en una cohorte de 48 niños positivos para COVID-19 ingresados en la UCI, la mayoría de quienes tenían comorbilidades previas (enfermedades genéticas incapacitantes en 40% de los casos) [32].

Una segunda explicación para la tendencia de los niños a ser menos afectados por el SARS-CoV-2 se relaciona con el receptor ACE2, que, como se mencionó previamente, se une al virus SARS-CoV-2. De hecho, una menor madurez y funcionalidad de la ACE2 y su menor expresión en el epitelio nasal en poblaciones pediátricas con respecto a los adultos podría explicar parcialmente la susceptibilidad reducida de los niños al COVID-19 [33, 34]. Sin embargo, los estudios no apoyan unilateralmente esta hipótesis. De hecho, algunos resultados sugieren que la expresión de ACE2 en niños no está aumentada ni disminuida [35, 36]. Por otro lado, un estudio encontró que la expresión de ACE2 disminuye una vez que el virus penetra en la célula y se replica, lo que reduce el número de receptores sobre los que el virus puede actuar [37]. A la luz de estos datos, aún se requiere conocer completamente el papel de los niveles de expresión de ACE2 en la patogénesis del daño pulmonar inducido por el COVID-19.

## Imágenes torácicas

El Colegio Norteamericano de Radiología recomienda realizar estudios radiológicos de imagen en pacientes pediátricos con diagnóstico confirmado de COVID-19 que presenten síntomas leves y comorbilidades preexistentes, y en niños con síntomas de moderados a graves. La radiografía torácica es el examen de primera elección; sin embargo, su menor sensibilidad y especificidad significa que no puede excluirse la afectación pulmonar en pacientes con un diagnóstico de COVID-19 confirmado en el laboratorio. A diferencia de lo que ocurre en adultos, la tomografía computarizada (TC) está indicada en niños en casos de sospecha de embolismo pulmonar y deterioro clínico [38].

### Radiografía torácica

Se encontraron anomalías pulmonares en 46–90% de los casos [39, 40]. Los signos radiológicos más comunes fueron engrosamiento peribronquial, opacidades con aspecto de vidrio molido, consolidación y efusión pleural [39]. Los hallazgos pulmonares fueron unilaterales en 55% de los niños afectados y bilaterales en 45%, sin diferencias significativas entre el pulmón derecho y el izquierdo, pero con mayor afectación de los lóbulos inferiores [40]. Aunque estas anomalías radiológicas se resuelven típicamente durante la recuperación de la enfermedad, se ha reportado que persisten en ∼16% de los casos [39].

### Tomografía computarizada torácica

Las anomalías radiológicas evidentes en TC son ciertamente mucho más descriptivas de la enfermedad, aunque estas anomalías son menos frecuentes y menos específicas que las descritas en adultos [41]. Los hallazgos más comunes son lesiones multifocales y de ubicación periférica, con apariencia de vidrio molido, que se inician en los lóbulos inferiores, acompañadas por engrosamiento en el septo interlobular, estructuras vasculares prominentes, signos del halo y signos del halo invertidos. En casos graves, se observa una llamativa apariencia de adoquinado y consolidación fragmentada [40, 41].

Liu y cols. describieron las características radiológicas que reveló la TC de alta resolución (high-resolution, HR) en cinco niños con un diagnóstico confirmado de COVID-19, tres de los cuales eran asintomáticos. Estos tres pacientes tenían opacidades redondas con aspecto de vidrio, mientras que solamente un niño mostró opacidad distribuida bilateralmente, y otro tuvo un reporte negativo [42]. Se resaltaron algunas diferencias radiológicas entre niños y adultos [43]. Aunque el hallazgo del aspecto de vidrio molido es característico y común tanto en la edad adulta y en la niñez, 44% de los pacientes adultos también tenían engrosamiento del septo interlobular, textura bronquial, un aspecto llamativo de adoquinado y − con menor frecuencia − signos del halo, efusión pleural o pericardial y linfadenopatía [44].

En un grupo de 98 pacientes de diversas edades (4–88 años) con COVID-19, la mayoría de las lesiones pulmonares visibles en TC-HR se localizaron en el lóbulo pulmonar inferior derecho, posiblemente debido a la estructura más fina y corta del bronquio en el lóbulo inferior, especialmente en el área periférica del pulmón. Sin embargo, niños y adolescentes tuvieron menos lesiones pulmonares, predominantemente con afectación unilateral, y menores cúmulos que los adultos, sin signos de broncograma aéreo [45]. Zheng y cols. también reportaron una mayor incidencia de deficiencia respiratoria en niños con edades < 3 años, con afectación pulmonar bilateral en > 70% de los niños en este grupo etario [46]. Un reporte de ocho pacientes con edades < 15 años ingresados en cuidados intensivos encontró anormalidades en TC exploratoria en todos los casos (seis niños con afectación bilateral, uno con afectación unilateral). Adicionalmente, dos de los ocho pacientes que originalmente tenían un peor pronóstico también mostraron una expresión mayor de IL-6 e IL-10, corroborando adicionalmente la relación entre la gravedad del cuadro pulmonar y la activación de la cascada de citocinas [47].

### Ultrasonido pulmonar

Varios estudios reportan la utilidad del ultrasonido pulmonar para el diagnóstico y seguimiento de neumonía por COVID-19, dado que es un estudio simple y reproducible que no expone al niño a la radiación ni a sedación. Entre los principales hallazgos ultrasonográficos que Musolino y cols. reportaron en 10 niños con COVID-19 se encuentran las líneas B (70%), irregularidades pleurales (60%), pulmón blanco (10%) y engrosamiento subpleural (10%) [48, 49]. De acuerdo con Allinovi y cols., el ultrasonido pulmonar puede apoyar el diagnóstico y monitoreo de neumonía por COVID-19, puesto que revela una pauta típica de síndrome pulmonar intersticial difuso y se correlaciona con los hallazgos de la TC torácica [50].

## Manejo y tratamiento de neumonía por COVID-19

Dado el curso paucisintomático característico de los niños diagnosticados con COVID-19, la mayoría de los casos sólo requieren terapia de soporte en casa. Evidentemente, los casos deben aislarse, y deben recibir cantidades adecuadas de fluidos y calorías [51, 52]. Para el manejo de la fiebre, se recomienda el paracetamol. Algunos autores han propuesto una correlación entre el uso de ibuprofeno y un curso más agresivo de infección por SARS-CoV-2 [53]; sin embargo, estos datos no se han confirmado. Para pacientes que ya recibían tratamiento con esteroides tópicos (p. ej., para la rinitis alérgica o el asma bronquial), está indicada la continuación de la terapia básica. En caso de que se requiera tratamiento inhalado con esteroides y broncodilatadores, se recomienda el uso de inhaladores presurizados con dosis predeterminadas con espaciador en vez de nebulizadores, que podrían aumentar la infectividad debido a la aerosolización de partículas [54].

La hospitalización está indicada cuando se requiere garantizar la terapia de soporte (p. ej., soporte farmacológico o respiratorio) o en formas graves de la patología [13, 19, 55]. Tras ingresar al hospital, puede ser útil practicar pruebas sanguíneas de laboratorio, aunque con frecuencia los resultados no son específicos. En la mayoría de los niños es posible encontrar: (i) un número normal o reducido de leucocitos, acompañado por linfocitopenia; (ii) valores normales o ligeramente aumentados de proteína C-reactiva y procalcitonina (en caso de un aumento excesivo en su expresión, debería considerarse la posibilidad de sobreinfección bacteriana); (iii) transaminasas y deshidrogenasas lácticas ligeramente aumentadas [13, 19, 55].

Debe prestarse mayor atención a los pacientes con enfermedades crónicas, porque la presencia de comorbilidades parece asociarse con un riesgo incrementado de evolución fatal (56). En este sentido, es necesario monitorear con mayor frecuencia a estos pacientes y brindarles tratamientos de forma más temprana.

### Soporte general

Es indispensable monitorear los signos vitales de los niños hospitalizados, y éstos deben recibir cantidades adecuadas de fluidos y calorías, con el propósito de mantener la homeostasis hidroelectrolítica. Adicionalmente, se recomienda guardar cama y mantener libres las vías respiratorias superiores [13, 19, 55].

### Terapia con oxígeno

En caso de hipoxia (SpO_2_ < 95%) sin signos de dificultad respiratoria, la administración de oxígeno mediante cánula nasal o máscara es suficiente, mientras se mantiene un monitoreo constante de los parámetros vitales y se atiende a los cambios en el balance ácido-base, que pueden ser indicativos de deterioro clínico [13, 19, 55].

### Soporte ventilatorio

En caso de dificultad respiratoria asociada con hipoxemia, la sola administración de oxígeno es insuficiente. En estos casos debe emplearse oxígeno nasal de alto flujo (HFNO) o ventilación no-invasiva, como presión positiva continua en las vías respiratorias (CPAP) [13, 19, 55]. La utilidad del HFNO para el tratamiento de COVID-19 aún es tema de debate, puesto que los incontrovertibles beneficios que brinda este tratamiento se contrarrestan por el riesgo de aerosolización de partículas virales en el entorno del paciente, poniendo por tanto en riesgo la seguridad de los trabajadores de salud [57]. La OMS recomienda emplear el HFNO en cuartos con un solo paciente o con presión negativa «siempre que sea posible». Esto significa que los cuartos con presión negativa, aunque deseables, no son esenciales [58]. Lo que sin duda es esencial, sin embargo, es el uso de equipo de protección personal (EPP) para entrar en el cuarto de los pacientes [57].

Una alternativa válida para el HFNO es la CPAP − de preferencia, CPAP con casco − con presión positiva al final de la espiración (PEEP) entre 5 y 10 cmH_2_0 (59). En cualquier caso, el niño críticamente enfermo debe transferirse a una unidad de cuidados intensivos pediátrica y, en el evento de la falta de respuesta a la ventilación no-invasiva o del inicio del síndrome pediátrico de dificultad respiratoria aguda (SDRA pediátrico), debe considerarse la posibilidad de poner al paciente en ventilación mecánica invasiva y, en última instancia, en oxigenación por membrana extracorpórea (ECMO) [19].

### Tratamiento farmacológico

Hay poca evidencia confiable sobre la utilidad de fármacos en el tratamiento de neumonía por COVID-19 en poblaciones pediátricas, y todos los datos disponibles hasta el momento se basan en observaciones hechas en poblaciones adultas. Por esta razón, no se aconseja administrar terapia farmacológica en las formas más leves de COVID-19, mientras que sí se recomienda en las formas más graves; invariablemente, la decisión debe tomarse caso por caso [13, 19, 51, 55, 60].

No se ha demostrado la efectividad de ningún fármaco específico contra el SARS-CoV-2. La terapia antiviral parece ser efectiva si se inicia antes del deterioro clínico. El fármaco más comúnmente utilizado es el interferón alfa administrado por nebulización, puesto que ha mostrado efectividad el reducir la replicación viral, con la consecuente mejoría en los síntomas y una menor duración de la enfermedad [21]. Otras posibles intervenciones farmacológicas incluyen:

Lopinavir/Ritonavir: Es una combinación de fármacos utilizada en el tratamiento del VIH que parece ser efectiva al reducir la replicación viral, siempre que se administre en etapas muy tempranas de la enfermedad. Los efectos colaterales comunes incluyen diarrea y náusea, y están contraindicados en casos de insuficiencia hepática [61].

Ribavirina: Es un fármaco utilizado en combinación con el interferón-alfa o el Lopnavir/Ritonavir. La anemia hemolítica es un posible efecto colateral [19, 62].

Remdesivir: Es un antiviral de nueva generación que tiene una potente acción antirreplicativa contra el SARS-CoV-2 [63, 64].

Hidroxicloroquina: Es un fármaco que, a pesar del entusiasmo inicial que causó su empleo para el tratamiento del COVID-19, no ha mostrado eficacia real de acuerdo con la evidencia científica más reciente [65]. La Tabla 2 presenta en forma sumaria los principales antivirales, sus formulaciones y las dosis respectivas en pacientes pediátricos.

Otros fármacos que vale la pena mencionar incluyen:

Antibióticos: No se recomienda su uso, a menos que haya signos de coinfección bacteriana. La utilidad de los macrólidos, especialmente la azitromicina, por sus propiedades antiinflamatorias, también es cuestionable [19, 52, 60, 66].

Corticosteroides: No se recomienda su uso rutinario; sin embargo, debe considerarse su empleo en casos de SDRA pediátrico, linfohistiocitosis hemofagocítica secundaria, choque séptico o asma concomitante. En estos casos, se recomienda la administración de metilprednisolona a una dosis de 1–2 mg/kg/día por un máximo de 4–5 días [19, 52, 60].

Gama globulinas: Su efectividad no está clara. Puede intentarse su empleo en formas particularmente graves de COVID-19 y en pacientes con síntomas similares a la enfermedad de Kawasaki, a una dosis de 2 g/kg/día por un día, 1 g/kg/día por dos días o 400 mg/kg/día por cinco días [67, 68].

Tocilizumab: Este anticuerpo monoclonal anti-IL-6 humano parecía ser efectivo en el tratamiento de adultos con afectación pulmonar extensa y bilateral [60]. Sin embargo, su eficacia se ha visto grandemente disminuida, al punto que no parece ser efectivo para prevenir la intubación o la muerte de pacientes hospitalizados moderadamente enfermos con COVID-19 [69]. Por esta razón, debe usarse con cautela en niños: 12 mg/kg en niños con peso <30 kg, 8 mg/kg (máx. 800 mg) en niños >30 kg, para repetir una vez después de 12 h si no hay mejoría [60, 70].

## Conclusión

En esta revisión se resumen las características del COVID-19 en poblaciones pediátricas, con énfasis en la afectación pulmonar. Aunque el cuadro clínico del COVID-19 en niños es mucho menos grave que en adultos, la progresión de la enfermedad sigue siendo posible y debe, por tanto, interceptarse con terapia apropiada. También debe enfatizarse que los niños, aunque paucisintomáticos, son vectores importantes de la enfermedad.

## Contribuciones de los autores

MM desarrolló la idea original e hizo la revisión final. GP escribió el manuscrito. CI y FD revisaron el manuscrito y contribuyeron a la revisión de la versión inglesa y a la compilación de referencias. SL hizo el análisis final y la revisión crítica del manuscrito. Todos los autores contribuyeron con el artículo y aprobaron la versión enviada.

## Conflicto de interés

Los autores declaran que la investigación se realizó en ausencia de cualquier relación comercial o financiera que pudiera considerarse un potencial conflicto de interés.

## Información sobre licencias

^©^ 2020 Guiseppe Fabio Parisi, Cristiana Indolfi, Fabio Decimo, Salvatore Leonardi, Michele Miraglia del Giudice: COVID-19 Pneumonia in Children: From Etiology to Management. Front Pediatr. 2020; 8: 616622 (traducción; contribuciones de los autores abreviada), con licencia bajo CC BY 4.0 (https://creativecommons.org/licenses/by/4.0/deed.es).

## Figures and Tables

**Tabla 1:** Clasificación de COVID-19 en niños

Clasificación	Características clínicas
Asintomático	Positividad del buffer RT-PCR para SARS-CoV-2 o serología positiva en ausencia de síntoma alguno de enfermedad.

Leve	Los síntomas son leves, y principalmente afectan las vías respiratorias superiores (obstrucción nasal, estornudos), a veces asociados con fiebre, tos y síntomas gastrointestinales.

Moderado	Los síntomas son más críticos; la fiebre y la tos (principalmente seca) casi siempre están presentes y se asocian con dificultades respiratorias. Se caracteriza radiológicamente por anomalías pulmonares compatibles con neumonía intersticial.

Grave	Se caracteriza por la presencia de hipoxemia (SpO_2_ <92%), con signos de dificultad respiratoria (taquipnea, gemido, aleteo, hundimiento), cianosis, signos y síntomas neurológicos, rechazo del alimento y signos de deshidratación.

Crítico	Progresión de la enfermedad con inicio de insuficiencia respiratoria que requiere ventilación mecánica, signos de choque o falla multiorgánica.

**Tabla 2:** Sumario de los antivirales más utilizados para el COVID-19 en niños

Antiviral	Forma de administración	Dosis pediátrica	Duración del tratamiento
Interferón-α* Lopinavir/Ritonavir	Inhalación Oral	200,000-400,000 Ul/kg en 2 mL de agua estéril, dos veces al día 12 mg/3 mg/kg si pesa 7–15 kg, 10 mg/2.5 mg/kg si pesa 15–40 kg, 400 mg/100 mg (dosis para adultos) si pesa >40 kg, dos veces al día	5–7 días 1–2 semanas
Ribavirina Remdesevir Hidroxicloroquina sulfato	Intravenoso Intravenoso Intravenoso	10 mg/kg/dosis, 2 o 3 veces al día dosis de carga de 5 mg/kg, posteriormente 2.5 mg/kg una vez al día −5 mg/kg/día (dosis máx. 400 mg), dos veces al día	máx. 5 días 10 días 5 días

* Usados más comúnmente.
